# Film or mirror? The exploration of narratives during the road from recognition to recovery of addictive disorders

**DOI:** 10.1192/j.eurpsy.2022.2112

**Published:** 2022-09-01

**Authors:** M. Krupa, E. Kiss, K. Kapornai

**Affiliations:** 1 University of Szeged Doctoral School, Educational Of Doctoral School, Szeged, Hungary; 2 University of Szeged, Department Of Pediatrics And Child Health Center, Szeged, Hungary

**Keywords:** addictive disorders, narrative of film, recovery, identity

## Abstract

**Introduction:**

The examination of the cinematic metanarrative provides many possibilities for recovery-oriented addiction consultation. The key to efficiency can be the approach of the recipient’s point of view and attitude, with which the client can interpret his own traumas and life story retrospectively.

**Objectives:**

Our aim is to show that the recognition, the turning points, the acknowledgement and the recovery from addiction can be described as a model in the deep structure of recovery stories. Can narrative research explore more deeply the main stages of recovery andidentity shaping, with the possible use of the film’s narrative technique?

**Methods:**

12 recovering addicts were interviewed who have been clean for at least 4 years. Interviews covered the years spent as addicts and the path to recovery using the method of deductive metanarrative analysis.

**Results:**

Based on the results of the analysis, elements of the film narrative could be found together major psychoanalysis concepts and literary theory models in the semi-structured interviews. Emotion control dysregulation all appear in the stories. Together these can be traced to a summary narrative and a historical line. Furthermore, the addicted person as a hero, the compulsion to repeat and its spookiness, and the role of the helpers also appear in the retrospective narratives without exception.

**Conclusions:**

The well-structured, coherent recovery stories help the recoverer to reconstruct their self, to make the behavioral change permanent, thus reducing the chances of relapse. The film narrative and toolkit provide an opportunity based on similarities with the narrator’s framework, which can strengthen the recovering identity.

**Disclosure:**

No significant relationships.

